# Editorial: Hippocampal mechanisms in aging and clinical memory decline

**DOI:** 10.3389/fnagi.2023.1204954

**Published:** 2023-05-05

**Authors:** Stephen D. Ginsberg, Stefano Tarantini

**Affiliations:** ^1^Center for Dementia Research, Nathan Kline Institute, Orangeburg, NY, United States; ^2^Department of Psychiatry, New York University Grossman School of Medicine, New York, NY, United States; ^3^Department of Neuroscience and Physiology, New York University Grossman School of Medicine, New York, NY, United States; ^4^NYU Neuroscience Institute, New York University Grossman School of Medicine, New York, NY, United States; ^5^Vascular Cognitive Impairment, Neurodegeneration and Healthy Brain Aging Program, Department of Neurosurgery, University of Oklahoma Health Sciences Center, Oklahoma City, OK, United States; ^6^Department of Health Promotion Sciences, College of Public Health, University of Oklahoma Health Sciences Center, Oklahoma City, OK, United States; ^7^The Peggy and Charles Stephenson Cancer Center, University of Oklahoma Health Sciences Center, Oklahoma City, OK, United States

**Keywords:** hippocampus, memory, aging, Alzheimer's disease, Alzheimer's disease related dementias

One of the most common cognitive problems that arises during the course of human senescence is a progressive decline in memory. Elders who otherwise live independent and robust lives often complain of forgetting or misplacing items as part of their daily activities. This is not a great revelation for any individual who interacts with an older family member, friend, or acquaintance. On face value, this may be considered one of the vagaries of aging. In fact, age-related memory loss has been the fodder of entertainment and literature for millennia. However, a deeper evaluation reveals many nuances regarding memory loss and potential opportunities to improve peoples' lives with mnemonic preservation.

Memory encompasses many modalities, including acquisition, encoding, processing, and recall, among others (Squire, 1986). Any one of these flash points can falter. There are short-term forms and long-term forms of memory. Not all aspects of memory are the same in terms of underlying structure, function, physiology, or mechanism of action. Accordingly, aging impacts what we broadly call “memory” in various ways. Some memory processing dysfunction is a common feature of what might be considered normal aging (Harada et al., 2013). However, progressive and pervasive memory decline that leads to loss of daily activity, social interactions, and deterioration of quality-of-life is associated with neurodegenerative disorders including Alzheimer's disease (AD) (Alzheimer's Association, 2023), is a devastating condition and a worldwide public health issue.

Rigorous and reproducible studies in live humans, postmortem human brains, and relevant animal models of aging and memory, including precise neuroanatomical tracing, neuroimaging, molecular, cellular, physiological, and behavioral studies have identified the hippocampal formation as the principal hub for mnemonic functions (Squire and Zola-Morgan, 1986; Rapp and Amaral, 1991; Huijgen and Samson, 2015). Not surprisingly, multiple components of the hippocampal formation are selectively vulnerable to cell, dendritic, and synapse loss as well as being a primary site for AD hallmark pathology, notably senile plaques (SPs) composed of amyloid-beta peptides and neurofibrillary tangles (NFTs) composed of the microtubule binding protein tau (Alzheimer's Association, 2023).

Hippocampal disconnection (Hyman et al., 1984, 1990) has been well established in the context of the onset and progression of AD (now recognized as an AD spectrum) (Jack et al., 2019a,b). Specifically, cell-type specific hippocampal formation neurodegeneration and localization of SPs and NFTs are profusely distributed throughout the AD hippocampus, and are an essential component of the neuropathological diagnosis of AD and Alzheimer's disease related dementias (ADRD) (Hyman and Trojanowski, 1997; Jack et al., 2019a,b). Neurodegeneration and neuron loss occurs throughout the hippocampal input-output circuitry. This is notable within stellate cells of the entorhinal cortex that project to the dentate gyrus, and layer IV entorhinal neurons that receive input from the subicular complex, which is the outflow network of the hippocampus (Gomez-Isla et al., 1996). Degeneration of CAl pyramidal cells and NFTs is a hallmark of AD-related pathology (Ginsberg et al., 1999). Subicular neurons also degenerate, and abundant NITs are found throughout the subiculum and parahippocampal gyrus (Ginsberg et al., 1999). Because the entire hippocampal formation circuit is critical for memory processing (Squire, 1986), hippocampal disconnection is a primary driver of the pervasive memory dysfunction seen in aging and neurodegenerative disorders.

The Research Topic “Hippocampal mechanisms in aging and clinical memory decline” was conceived to highlight basic and translational research in human hippocampus and relevant animal models aiming to provide evidence-based scientific inquiry into mechanisms that drive memory loss in aging and AD/ADRD.

In an original article, Matuskova et al., designed a series of experiments to interrogate underlying structural drivers of neuropsychiatric symptoms (NPS) often associated with pathological aging, including AD, in a healthy aging memory clinic cohort of elders employing the Mild Behavioral Impairment Checklist (MBI-C) tool. The authors found the entorhinal cortex was associated with total score MBI-C performance. Moreover, in this nondemented cohort, NPS is associated with medial temporal lobe atrophy, which indicates the MBI-C may be useful for early identification of individuals with NPS who are at a higher risk for dementia.

Leveraging recent findings that Vang-Gogh like 2 (Vangl2), a key constituent of the planar cell polarity (PCP) signaling pathway, is localized to the adult murine dentate gyrus, Koehl et al., in an original article investigated the impact of Vangl2 on neurogenesis and memory in middle aged mice. Through the study of a Vangl2 mutant model construct, the authors demonstrate normal Vangl2 expression impacts granule cells born in adults and is a key regulator of cognitive flexibility. These provocative preclinical results suggest targeting Vangl2 may be useful in attenuating age-related memory decline.

In a systematic review, Zhou et al., evaluated five exercise regimens, aerobic training, combined training, high-intensity interval training, resistance training, and aerobic training plus resistance training, on serum levels of the neurotrophin brain-derived neurotrophic factor (BDNF) in healthy aging and diseased cohorts. Bayesian network meta-analysis of 39 randomized controlled trials with 2,031 participants rank ordered resistance training as the optimal exercise approach for both children and elders in terms of sustaining serum BDNF levels to support CNS-related benefits.

Mukli et al., in an original article, tested the hypothesis that gait variability and gait asymmetry are reliable predictors of cognitive decline in elders with cerebral small vessel disease (CSVD). Cognitive performance, as measured by the Cambridge Neuropsychological Test Automated Battery (CANTAB) and gait were evaluated in a cohort of elders with CSVD. Results indicate that step lengths in individuals with CSVD had greater asymmetry than controls. Moreover, gait variability had an inverse association with sustained attention, most notably in individuals with CSVD. These data are supportive of the concept that gait variability and asymmetry are viable predictors of cognitive decline in elders with CSVD.

The evidence presented in these articles complements the existing body of knowledge in the literature (Santín-Márquez et al., 2021; Kilimann et al., 2022; Kiss et al., 2022; Milicic et al., 2022; Zhang et al., 2022) by providing further insight into the hippocampal mechanisms of memory decline in aging and neurodegenerative disorders such as AD. Specifically, this Research Topic highlighted the critical role of the hippocampal formation in memory processing and the selective vulnerability of this brain region to neurodegeneration, synaptic loss, and the accumulation of hallmark pathologies such as SPs and NFTs. These studies provide new evidence and insights into the mechanisms of memory decline in aging and neurodegenerative disorders and potential interventions for improving cognitive function in older adults.

A general caveat regarding this Research Topic, “Hippocampal mechanisms in aging and clinical memory decline” is that it is not possible to articulate all of the basic, translational, and clinical research components driving memory decline in aging in the context of a circumscribed set of manuscripts on this broad topic. Rather, a stated goal of the Research Topic series in *Frontiers in Aging Neuroscience* is to increase awareness and interest in an underserved area with significant public health importance to the aging community writ large. It is our hope this special issue drives greater interest and encourages new investigators from other scientific backgrounds to begin studying aspects of memory dysfunction in aging and AD/ADRD.

## Author contributions

SDG and ST wrote and edited the manuscript and take responsibility data integrity of and the accuracy presented herein. Both authors contributed to the article and approved the submitted version.
